# Health comorbidities and cognitive abilities across the lifespan in Down syndrome

**DOI:** 10.1186/s11689-019-9306-9

**Published:** 2020-01-23

**Authors:** Carla M. Startin, Hana D’Souza, George Ball, Sarah Hamburg, Rosalyn Hithersay, Kate M. O. Hughes, Esha Massand, Annette Karmiloff-Smith, Michael S. C. Thomas, LonDownS Consortium, André Strydom, Elizabeth Fisher, Dean Nizetic, John Hardy, Victor Tybulewicz, Annette Karmiloff-Smith, Michael Thomas, Denis Mareschal, Tamara Al-Janabi, Nidhi Aggarwal, Tommy Coyle, Amy Davies, Lucy Fodor-Wynne, Bryony Lowe, Erin Rodger, Joanne Ball, Dan Brady, Amelia Channon, Charlotte Dennison, Maria Eriksson, Amanda Lathan, Beejal Mehta, Lidija Nikolova, Jessica Schulz, Laura Simmons, Margaux Trombert, Kate Whitaker, John Hardy, Kin Y. Mok, David Zhang, Andre Strydom

**Affiliations:** 1 Department of Forensic and Neurodevelopmental Sciences, Institute of Psychiatry, Psychology and Neuroscience, King’s College London, London, UK; 2 Division of Psychiatry, University College London, London, UK; 3 The LonDownS Consortium (London Down Syndrome Consortium), London, UK; 4 Centre for Brain and Cognitive Development, Birkbeck, University of London, London, UK; 5 Department of Psychology & Newnham College, University of Cambridge, Cambridge, UK; 6 Department of Psychology, University of Arizona, Tucson, AZ USA; 7 South London and the Maudsley NHS Foundation Trust, London, UK

**Keywords:** Down syndrome, Intellectual disability, Health comorbidities, Psychiatric comorbidities, Receptive language ability, Cognitive outcomes

## Abstract

**Background:**

Down syndrome (DS) is associated with variable intellectual disability and multiple health and psychiatric comorbidities. The impact of such comorbidities on cognitive outcomes is unknown. We aimed to describe patterns of physical health and psychiatric comorbidity prevalence, and receptive language ability, in DS across the lifespan, and determine relationships with cognitive outcomes.

**Methods:**

Detailed medical histories were collected and cognitive abilities measured using standardised tests for 602 individuals with DS from England and Wales (age range 3 months to 73 years). Differences in prevalence rates between age groups and between males and females were determined using chi-squared or Fisher’s exact tests. In adults, rates for psychiatric comorbidities were compared to expected population rates using standardised morbidity ratios (SMRs). Adapted ANCOVA functions were constructed to explore age and sex associations with receptive language ability across the lifespan, and regression analyses were performed to determine whether the presence of health comorbidities or physical phenotypes predicted cognitive abilities.

**Results:**

Multiple comorbidities showed prevalence differences across the lifespan, though there were few sex differences. In adults, SMRs were increased in males and decreased in females with DS for schizophrenia, bipolar disorder, and anxiety. Further, SMRs were increased in both males and females with DS for dementia, autism, ADHD, and depression, with differences more pronounced in females for dementia and autism, and in males for depression. Across the lifespan, receptive language abilities increasingly deviated from age-typical levels, and males scored poorer than females. Only autism and epilepsy were associated with poorer cognitive ability in those aged 16–35 years, with no relationships for physical health comorbidities, including congenital heart defects.

**Conclusions:**

Our results indicate the prevalence of multiple comorbidities varies across the lifespan in DS, and in adults, rates for psychiatric comorbidities show different patterns for males and females relative to expected population rates. Further, most health comorbidities are not associated with poorer cognitive outcomes in DS, apart from autism and epilepsy. It is essential for clinicians to consider such differences to provide appropriate care and treatment for those with DS and to provide prognostic information relating to cognitive outcomes in those with comorbidities.

## Background

Down syndrome (DS), caused by chromosome 21 triplication, is the most common genetic cause of intellectual disability (ID), with a UK incidence of approximately one in 1000 live births [1]. DS is associated with a distinct phenotype involving many body systems. ID across the lifespan and the development of dementia in later life are almost universal in people with DS [2, 3], with a suggested cumulative incidence of dementia of 95.7% by age 68 [4]. A number of other health phenotypes are associated with DS, including short stature, microcephaly, congenital heart defects, endocrine disorders (in particular hypothyroidism), higher risk for infections, and obstructive sleep apnoea [5–10]. DS is also associated with increased risk for other neurodevelopmental conditions including autism and attention deficit hyperactivity disorder (ADHD) [11]. There is however considerable variability in both health comorbidities and degree of intellectual impairment among people with DS [2, 12], and links between health comorbidities and cognitive outcomes are not currently well understood.

Such associations between health comorbidities and cognitive outcomes may be due to a hypothesised effect of a health phenotype or comorbidity on cognitive outcomes (for example, in the typically developing population, congenital heart defects have been associated with poorer cognitive abilities [13]), or shared genetic mechanisms between health and cognitive phenotypes. Understanding such associations may be informative for prognosis for those with DS.

Further, patterns of comorbidity rates in DS may vary over the lifespan due to improvements in care and treatment and between males and females, and so detailed up-to-date information is required to inform clinical services, individuals, and their families and carers. To provide a current understanding of health comorbidities, including psychiatric comorbidities, in individuals with DS and their potential relationships with cognitive outcomes, we conducted one of the largest cross-sectional studies of individuals with DS to date. We focused on early childhood (i.e., up to age 5) as this is a critical period for development, and adulthood (i.e., over age 16) to explore changes associated with ageing. We had two main aims: firstly to describe patterns of the prevalence of common health comorbidities across the lifespan, also considering sex differences in rates for psychiatric comorbidities relative to expected general population rates, and secondly to explore relationships between receptive language ability and general cognitive abilities with age and health comorbidities, respectively.

## Methods

### Participants

Between 2013 and 2016, 605 individuals with a clinical diagnosis of DS mainly across England and Wales were recruited via DS support groups, existing participant databases, care homes, and National Health Service Trust sites in four age groups: younger children (3 months to 5.5 years), older children (5.5 to 15 years), younger adults (16 to 35 years), and older adults (36+ years). Age groups were defined based on previous definitions of life stages in DS [14]. Clinical diagnosis of DS was confirmed genetically using saliva or blood samples where possible (see Table 1); following DNA extraction, genome-wide single nucleotide polymorphism genotyping was performed using an Illumina OmniExpressExome array (San Diego, CA, USA) at UCL Genomics, then assembled and visually inspected in GenomeStudio to confirm the presence of chromosome 21 trisomy, mosaicism, or partial trisomy. Three adults aged 36+ were excluded from further analyses after genetic analysis did not suggest an additional chromosome 21, leaving 602 participants.

**Table 1:** Participant demographic information, the prevalence of selected health comorbidities in each age group, and prevalence comparisons

			Younger children (0–5.5 years)	Older children (5.5–15 years)	Younger adults (16–35 years)	Older adults (36+ years)	Younger adults vs younger children	Older adults vs younger adults
Demographic information	Number		115	35	170	282	N/A	N/A
	Age		2.18 ± 1.13 (3.6 months to 5 years 1.3 months)	10.63 ± 3.05 (5 years 6.5 months to 14 years 8.5 months)	25.25 ± 5.46 (16–35 years)	50.21 ± 7.76 (36–73 years)	N/A	N/A
	Sex	Male	62 (53.9%)	16 (45.7%)	83 (48.8%)	153 (54.3%)	N/A	N/A
		Female	53 (46.1%)	19 (54.3%)	87 (51.2%)	129 (45.7%)	N/A	N/A
	Ethnicity	White	93 (81.6%)	25 (86.2%)	145 (85.3%)	261 (92.6%)	N/A	N/A
		Black	5 (4.4%)	0 (0.0%)	7 (4.1%)	11 (3.9%)	N/A	N/A
		Asian	6 (5.3%)	0 (0.0%)	8 (4.7%)	6 (2.1%)	N/A	N/A
		Mixed	7 (6.1%)	4 (13.8%)	8 (4.7%)	2 (0.7%)	N/A	N/A
		Other	3 (2.6%)	0 (0.0%)	2 (1.2%)	2 (0.7%)	N/A	N/A
	Parental socioeconomic status^a^	1	24 (22.6%)	9 (27.3%)	21 (16.0%)	16 (9.5%)	N/A	N/A
		2	47 (44.3%)	14 (42.4%)	55 (42.0%)	66 (39.1%)	N/A	N/A
		3	19 (17.9%)	5 (15.2%)	23 (17.6%)	10 (5.9%)	N/A	N/A
		4	5 (4.7%)	1 (3.0%)	15 (11.5%)	13 (7.7%)	N/A	N/A
		5	4 (3.8%)	2 (6.1%)	8 (6.1%)	36 (21.3%)	N/A	N/A
		6	3 (2.8%)	1 (3.0%)	6 (4.6%)	4 (2.4%)	N/A	N/A
		7	2 (1.9%)	1 (3.0%)	0 (0.0%)	5 (3.0%)	N/A	N/A
		8	1 (0.9%)	0 (0.0%)	2 (1.5%)	9 (5.3%)	N/A	N/A
		9	1 (0.9%)	0 (0.0%)	1 (0.8%)	10 (5.9%)	N/A	N/A
		Missing	9	2	39	113	N/A	N/A
	DS type^a^	Trisomy 21	93 (96.9%)	26 (96.3%)	157 (95.7%)	250 (96.5%)	N/A	N/A
		Mosaic	2 (2.1%)	0 (0.0%)	4 (2.4%)	6 (2.3%)	N/A	N/A
		Partial trisomy	1 (1.0%)	1 (3.7%)	3 (1.8%)	3 (1.2%)	N/A	N/A
		Unknown	19	8	6	23	N/A	N/A
Physical measurements	Height (cm)		81.21 ± 9.72	N/A	152.44 ± 10.32	150.11 ± 9.92	N/A	N/A
	Weight (kg)		11.62 ± 2.80	N/A	69.78 ± 16.14	68.17 ± 16.09	N/A	N/A
	BMI		N/A	N/A	30.09 ± 7.01	30.47 ± 7.25	N/A	N/A
	Head circumference (cm)		46.02 ± 2.26	50.40 ± 2.15	53.45 ± 2.55	52.57 ± 2.28	N/A	N/A
Psychiatric	Autism		N/A	2 (5.7%)	23 (13.5%) ^+^	7 (2.5%) ^+^	N/A	OR = 0.16 (0.07, 0.39), *Χ*(1) = 20.89, *p* < 0.001^b^
	ADHD		N/A	3 (8.6%)	5 (2.9%) ^+^	1 (0.4%) ^+^	N/A	OR = 0.12 (0.01, 1.01), *p* = 0.030^c^
	Schizophrenia		N/A	0 (0.0%)	1 (0.6%)	3 (1.1%)	N/A	OR = 1.82 (0.19, 17.61), *p* = 1.000^c^
	Bipolar disorder		N/A	1 (2.9%)	1 (0.6%)	6 (2.1%)	N/A	OR = 3.67 (0.44, 30.78), *p* = 0.263^c^
	Depression		N/A	0 (0.0%)	21 (12.4%)	52 (18.4%)	N/A	OR = 1.60 (0.93, 2.77), *Χ*(1) = 2.90, *p* = 0.088^b^
	Anxiety		N/A	1 (2.9%)	14 (8.2%)	18 (6.4%)	N/A	OR = 0.76 (0.37, 1.57), *Χ*(1) = 0.55, *p* = 0.457^b^
Neurological	Cerebral palsy		0 (0.0%)	1 (2.9%)	1 (0.6%)	0 (0.0%)	*p* = 1.000^c^	*p* = 0.376^c^
	Dementia		N/A	N/A	0 (0.0%) ^+^	90 (31.9%) ^+^	N/A	*Χ*(1) = 67.74, *p* < 0.001^b^
	Parkinson’s disease		N/A	N/A	0 (0.0%)	0 (0.0%)	N/A	N/A
	Stroke		N/A	N/A	0 (0.0%)	2 (0.7%)	N/A	*p* = 0.530^c^
	Migraine		N/A	N/A	2 (1.2%)	2 (0.7%)	N/A	OR = 0.60 (0.08, 4.30), *p* = 0.634^c^
	Epilepsy / seizures		5 (4.3%)	5 (14.3%)	17 (10.0%) ^+^	58 (20.6%) ^+^	OR = 2.44 (0.88, 6.83), *Χ*(1) = 3.08, *p* = 0.079^b^	OR = 2.33 (1.31, 4.16), *Χ*(1) = 8.56, *p* = 0.003^b^
	Insomnia		0 (0.0%) *	0 (0.0%)	9 (5.3%) *	16 (5.7%)	*p* = 0.012^c^	OR = 1.08 (0.47, 2.49), *Χ*(1) = 0.03, *p* = 0.864^b^
Physical health	Obstructive sleep apnoea		1 (0.9%) *	2 (5.7%)	27 (15.9%) * ^+^	11 (3.9%) ^+^	OR = 21.52 (2.88, 160.81), *Χ*(1) = 17.45, *p* < 0.001^b^	OR = 0.22 (0.10, 0.45), *Χ*(1) = 19.77, *p* < 0.001^b^
	Congenital heart defects	Total	63 (54.8%)	19 (54.3%)	78 (45.9%) ^+^	49 (17.4%) ^+^	OR = 0.70 (0.44, 1.13), *Χ*(1) = 2.17, *p* = 0.140^b^	OR = 0.25 (0.16, 0.38), *Χ*(1) = 42.66, *p* < 0.001^b^
		Known AVSD	49 (42.6%)	12 (34.3%)	36 (21.2%)	9 (3.2%)	N/A	N/A
		Surgery	22 (19.1%)	5 (14.3%)	36 (21.2%) ^+^	6 (2.1%) ^+^	OR = 1.14 (0.63, 2.05), *Χ*(1) = 0.18, *p* = 0.674^b^	OR = 0.08 (0.03, 0.20), *Χ*(1) = 45.66, *p* < 0.001^b^
	Recurrent pneumonia		5 (4.3%)	3 (8.6%)	9 (5.3%)	13 (4.6%)	OR = 1.23 (0.40, 3.77), *Χ*(1) = 0.13, *p* = 0.717^b^	OR = 0.87 (0.36, 2.07), *Χ*(1) = 0.11, *p* = 0.743^b^
	Coeliac disease		0 (0.0%)	2 (5.7%)	4 (2.4%)	5 (1.8%)	*p* = 0.150^c^	OR = 0.75 (0.20, 2.83), *p* = 0.734^c^
	Rheumatoid arthritis		N/A	N/A	0 (0.0%)	6 (2.1%)	N/A	*p* = 0.088^c^
	Psoriasis		0 (0.0%) *	0 (0.0%)	7 (4.1%) *	12 (4.3%)	*p* = 0.044^c^	OR = 1.04 (0.40, 2.68), *Χ*(1) = 0.01, *p* = 0.944^b^
	Eczema		11 (9.6%) *	6 (17.1%)	2 (1.2%) *	7 (2.5%)	OR = 0.11 (0.02, 0.52), *Χ*(1) = 11.09, *p* = 0.001^b^	OR = 2.14 (0.44, 10.41), *p* = 0.494^c^
	Gout		N/A	N/A	2 (1.2%)	11 (3.9%)	N/A	OR = 3.41 (0.75, 15.57), *p* = 0.144^c^
	Hypothyroid		8 (7.0%) *	3 (8.6%)	52 (30.6%) * ^+^	117 (41.5%) ^+^	OR = 5.89 (2.68, 12.97), *Χ*(1) = 23.05, *p* < 0.001^b^	OR = 1.61 (1.08, 2.41), *Χ*(1) = 5.38, *p* = 0.020^b^
	Hyperthyroid		3 (2.6%)	1 (2.9%)	2 (1.2%)	2 (0.7%)	OR = 0.44 (0.07, 2.70), *p* = 0.396^c^	OR = 0.60 (0.08, 4.30), *p* = 0.634^c^
	Diabetes	Type 1	0 (0.0%)	0 (0.0%)	1 (0.6%)	3 (1.1%)	*p* = 1.000^c^	OR = 1.82 (0.19, 17.61), *p* = 1.000^c^
		Type 2	0 (0.0%)	0 (0.0%)	0 (0.0%) ^+^	8 (2.8%) ^+^	N/A	*p* = 0.027^c^
	Reflux		39 (33.9%) *	15 (42.9%)	5 (2.9%) *	14 (5.0%)	OR = 0.06 (0.02, 0.16), *Χ*(1) = 50.40, *p* < 0.001^b^	OR = 1.72 (0.61, 4.87), *Χ*(1) = 1.08, *p* = 0.299^b^
	Leukaemia		0 (0.0%)	1 (2.9%)	4 (2.4%) ^+^	0 (0.0%) ^+^	*p* = 0.150^c^	*p* = 0.020^c^
	Cancerous solid tumours		N/A	N/A	0 (0.0%)	1 (0.4%)	N/A	*p* = 1.000^c^
Vision and hearing	Vision impairments^a^		27 (23.5%) *	10 (28.6%)	128 (77.6%) *	191 (75.5%)	OR = 11.28 (6.41, 19.85), *Χ*(1) = 80.25, *p* < 0.001^b^	OR = 0.89 (0.56, 1.42), *Χ*(1) = 0.24, *p* = 0.625^b^
	Cataracts		N/A	N/A	15 (8.8%) ^+^	76 (27.0%) ^+^	N/A	OR = 3.81 (2.11, 6.89), *Χ*(1) = 21.67, *p* < 0.001^b^
	Glaucoma		N/A	N/A	0 (0.0%)	4 (1.4%)	N/A	*p* = 0.302^c^
	Hearing impairments^a^		32 (27.8%) *	11 (31.4%)	26 (16.4%) * ^+^	74 (29.6%) ^+^	OR = 0.51 (0.28, 0.91), *Χ*(1) = 5.27, *p* = 0.022^b^	OR = 2.15 (1.30, 3.55), *Χ*(1) = 9.23, *p* = 0.002^b^
	Otitis media with effusion (glue ear)		64 (55.7%) *	26 (74.3%)	44 (25.9%) * ^+^	15 (5.3%) ^+^	OR = 0.28 (0.17, 0.46), *Χ*(1) = 25.83, *p* < 0.001^b^	OR = 0.16 (0.09, 0.30), *Χ*(1) = 39.52, *p* < 0.001^b^

Values for age and physical measurements show mean ± standard deviation. Parental socioeconomic status (SES) groups are as follows: 1 managers / directors / senior officials, 2 professional occupations, 3 associate professional and technical occupations, 4 administrative and secretarial occupations, 5 skilled trade occupations, 6 caring, leisure, and other service occupations, 7 sales and customer service occupations, 8 process, plant, and machine operatives, 9 elementary occupations.

*ADHD* attention deficit hyperactivity disorder, *AVSD* atrioventricular septal defect, *N/A* not applicable, *OR* odds ratio

* Significant difference in prevalence between younger children and younger adults (*p* < 0.05), ^+^ significant difference in prevalence between younger adults and older adults (*p* < 0.05).

^a^ Percentages calculated based on subsamples; for SES and DS type this excluded individuals whose SES / DS type was unknown, for vision impairments younger adults *n* = 165 and older adults *n* = 253, for hearing impairments younger adults *n* = 159 and older adults *n* = 250. Where prevalence is N/A information was not included in medical questionnaire. Values for comparisons give odds ratios (95% CIs; not possible where one cell equals zero), and statistical comparisons performed using ^b^ chi-squared tests or ^c^ Fisher’s exact test as appropriate.

### Ethical approval

Ethical approval was obtained for all adults and younger children from the North West Wales Research Ethics Committee (13/WA/0194), and for younger and older children from the Birkbeck College Ethics Committee (121373 and 151632, respectively). Written informed consent was obtained from the parents of all children, from adults where they had capacity to consent, and via an appointed consultee where adults did not have capacity to consent, in accordance with the UK Mental Capacity Act 2005.

### Demographic information

Participants’ basic demographic information was obtained via parental or carer report. Socioeconomic status (SES) was determined based on maternal and paternal occupations, using the highest major group for the two occupations as classified by the UK Office of National Statistics standard occupational classification 2010 (possible score range 1–9 with lower scores representing higher SES).

### Assessment of health phenotypes

#### Physical measurements

Height, weight, and head circumference were measured where possible; body mass index (BMI) was calculated for adults. BMI measurements are not recommended for use in younger children, so were not calculated for this group.

#### Medical histories

Participants’ detailed lifetime medical histories, consisting of current and previous clinical diagnoses, were collected via informant report from caregivers. These were confirmed with carer-held medical records where possible. Semi-structured interviews were conducted over the telephone with parents for children, and face-to-face with a relative (37.6% parent, 12.2% other relative) or paid carer (50.2%) for adults, via a checklist of conditions (see Table 1). Where possible, medical histories collected from paid carers were verified with a relative over the telephone to ensure accurate information on conditions in early life.

### Measures of cognitive ability

Cognitive abilities were assessed using age-appropriate measures by trained researchers, usually in participants’ homes for adults and using our testing rooms for children.

Younger children were administered the *Mullen Scales of Early Learning (MSEL)* [15] to assess developmental abilities across five subscales: receptive language, expressive language, visual reception, gross motor, and fine motor abilities. Receptive language ability scores were used in lifespan analyses.

Older children were administered the *British Picture Vocabulary Scale 3 (BPVS3)* [16] to assess receptive language abilities.

Younger and older adults who met vision and hearing screening thresholds (3/19 on the Kay vision test and a loud voice on the Whisper test, see Startin et al. [2]) were administered the *Kaufmann Brief Intelligence Test 2 (KBIT-2)* [17]. The KBIT-2 assesses receptive language verbal abilities and non-verbal abilities. Adults with adequate vision and hearing who were unable to attempt tasks due to the severity of their cognitive impairment or presence of dementia were given a score of zero (*n* = 34, 8.6%). Receptive language verbal ability scores were used in lifespan analyses.

### Statistical analysis

Prevalence rates of health comorbidities were calculated for each age group. To identify changes in prevalence across the lifespan, rates were compared between younger children and younger adults, and between younger adults and older adults. To identify sex differences in prevalence, rates for males and females were compared for younger children, younger adults, and older adults separately. Prevalence comparisons used chi-squared tests or Fisher’s exact test as appropriate. These analyses did not include older children due to the smaller participant numbers.

For psychiatric comorbidities and dementia, standardised morbidity ratios (SMRs) for adults were estimated using the indirect method by comparing our observed prevalence rates to expected UK general population rates from Prince et al. [18] for dementia and McManus et al. [19] for other comorbidities. Splitting analysis by sex, observed and expected rates were calculated in 10-year age bands and then summed. Dividing observed rates by expected rates provided SMRs for comparison between populations (see Additional file 1: Tables S1a and S1b). SMR confidence intervals (CIs) were obtained using exact 95% Poisson CIs.

Age-adjusted *z*-scores for available raw receptive language scores were calculated from the means and standard deviations given in standardised tables for each cognitive test. A *z*-score of 0 corresponds to age-typical performance, while a *z*-score of − 1 corresponds to performance one standard deviation below this. Based on previous findings of significant differences between receptive language *z*-scores calculated for the KBIT-2 and BPVS3, BPVS3 raw scores for older children were first converted to KBIT-2 verbal raw scores using a linear interpolation method based on the relationship between the two scores from a sub-sample of adults who had completed both tests (*r* = 0.96, *p* < 0.001, *n* = 34) [20]. We therefore used age-typical levels for KBIT-2 verbal scores to determine *z*-scores for older children and all adults, and age-typical levels from the MSEL receptive language subscale to determine *z*-scores for younger children. Adapted ANCOVA functions were then constructed across all ages for receptive language *z*-scores (*n* = 523) and for each age group separately for raw receptive language scores (younger children *n* = 104, older children *n* = 25, younger adults *n* = 157, older adults *n* = 237) to determine associations with age and sex, and their interaction, with associated effect sizes determined using *η*_*p*_^2^. All analyses used age as a continuous variable with sex as two groups.

To examine whether the presence of health comorbidities (using a threshold of a minimum prevalence of 10%) or physical phenotypes predicted cognitive abilities, separate multiple regression analyses were performed for younger children (*n* = 99) using mean MSEL raw scores calculated from subscale raw scores excluding the gross motor scale (which does not go beyond 33 months) and younger adults (*n* = 157) using KBIT-2 total raw scores (sum of verbal and non-verbal scores). Raw scores were used due to floor effects when converting to standardised scores. Analyses were not performed for older adults due to the known risk of cognitive decline due to dementia, or for older children due to the smaller participant numbers. Hierarchical regression analyses (Enter method) were conducted. Model 1 contained sex, age (in years to two decimal places for younger children and full years for younger adults), and SES. Model 2 added the health comorbidity or physical phenotype of interest to determine whether a further significant proportion of variance in cognitive ability was explained. Where a comorbidity or phenotype explained a significant proportion of variance, additional regressions were performed using raw subscale scores separately in Model 2, to assess specificity of effects.

Analyses were conducted using SPSS, with *p* < 0.05 indicating statistical significance, aside from SMRs, which were determined as described above.

## Results

Age, sex, ethnicity, physical measurements, and the observed prevalence of health comorbidities for 115 younger children, 35 older children, 170 younger adults, and 282 older adults are shown in Table 1.

### Health comorbidities across the lifespan

Eczema, reflux, hearing impairments, and otitis media with effusion (glue ear) were reported to be more common in younger children compared to younger adults, and insomnia, obstructive sleep apnoea, psoriasis, hypothyroidism, and vision impairments less common. Autism, ADHD, obstructive sleep apnoea, congenital heart defects and related surgery, history of leukaemia, and otitis media with effusion were reported to be more common in younger adults compared to older adults, and dementia, epilepsy, hypothyroidism, type 2 diabetes, cataracts, and hearing impairments less common (Table 1).

### Sex differences in prevalence rates of health comorbidities

Several statistically significant sex differences in prevalence of health comorbidities were observed (Table 2). Higher rates were reported in males compared to females for otitis media with effusion in younger children and older adults, and for reflux in younger adults. Higher rates were reported in females compared to males for hypothyroidism in older adults. No other sex comparisons were significant, including for psychiatric comorbidities (all *p* > 0.05).

**Table 2:** Significant differences in health comorbidity prevalence between males and females

	Males	Females	Statistical comparison
Younger children—otitis media with effusion	40 (64.5%)	24 (45.3%)	OR = 2.20 (1.04, 4.65), *X*(1) = 4.28, *p* = 0.039^a^
Younger adults—reflux	5 (6.0%)	0 (0.0%)	*p* = 0.026^b^
Older adults—hypothyroidism	55 (35.9%)	62 (48.1%)	OR = 0.61 (0.38, 0.98), *X*(1) = 4.23, *p* = 0.040^a^
Older adults—otitis media with effusion	12 (7.8%)	3 (2.3%)	OR = 3.57 (0.99, 12.96), *X*(1) = 4.23, *p* = 0.040^a^

Results show the prevalence in males and females (*n* (%)) with results of statistical analysis giving odds ratios (95% CIs; not possible where one cell equals zero), and statistical comparisons performed using ^a^ chi-squared tests or ^b^ Fisher’s exact test as appropriate.

*OR* odds ratio

### Prevalence of psychiatric comorbidities compared to population rates

For adults, SMRs indicated rates of dementia, autism, ADHD, and depression were higher in individuals with DS compared to population rates. For dementia and autism, this relationship was significantly more pronounced for females than males. For depression, this relationship was significantly more pronounced for males than females. Schizophrenia, bipolar disorder, and anxiety had higher rates in males with DS relative to male population rates, while these comorbidities had lower rates in females with DS relative to female population rates (Table 3).

**Table 3:** Standardised morbidity ratios (SMRs) comparing prevalence rates in adults with DS to UK population rates

	SMR males	SMR females
Autism	6.83 (6.04, 7.69)	17.60 (14.78, 20.67)
ADHD	5.04 (4.06, 6.27)	5.56 (4.50, 6.84)
Schizophrenia	3.67 (3.14, 4.27)	0.49 (0.33, 0.68)
Bipolar disorder	1.22 (1.05, 1.42)	0.79 (0.65, 0.95)
Depression	4.97 (4.66, 5.29)	3.97 (3.72, 4.23)
Anxiety	1.75 (1.61, 1.90)	0.57 (0.50, 0.64)
Dementia	43.33 (41.14, 45.58)	50.52 (48.13, 52.99)

Figures show SMRs (95% CI) adjusted for age and sex. Population rates for dementia were taken from Prince et al. [18], all other rates taken from McManus et al. [19]. If the CI for the SMR includes 1, there is no significant difference in rates for adults with DS and population rates. An SMR lower than 1 indicates the prevalence in adults with DS is lower than UK population rates. A value higher than 1 indicates the prevalence in adults with DS is higher than UK population rates. Non-overlapping CIs in SMRs for males and females indicate a significant sex difference relative to the population difference.

*ADHD* attention deficit hyperactivity disorder

### Receptive language across the lifespan

**Fig. 1:**
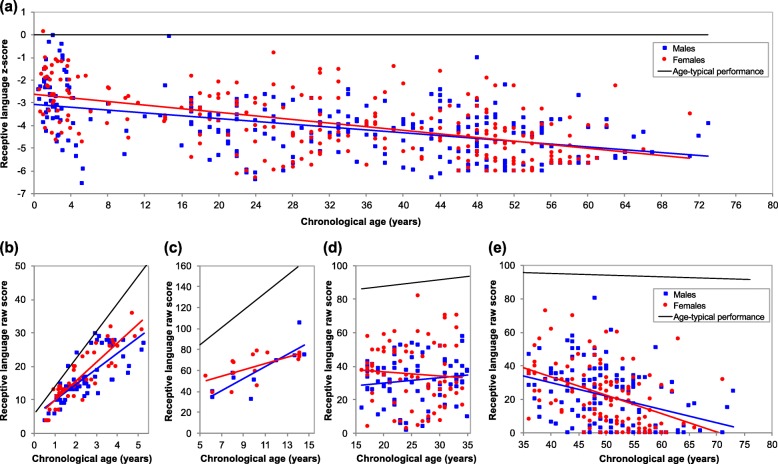
Changes in receptive language ability across the lifespan in DS. Lines show performance for males (blue) and females (red), with age-typical performance (black). The top graph (**a**) represents receptive language *z*-scores across the lifespan (males *n* = 271, females *n* = 252), with a value of 0 corresponding to age-typical performance. The bottom graphs (**b-e**) represent raw scores coresponding to the *z*-scores in the top graph, split into scores for younger children (**b**; males *n* = 59, females *n* = 45), older children (**c**; males *n* = 10, females *n* = 15), younger adults (**d**; males *n* = 80, females *n* = 77), and older adults (**e**; males *n* = 122, females *n* = 115). Children with DS develop abilities (**b** and **c**) but do so at a slower pace than typically developing children, as reflected by a decrease in *z*-scores over childhood (**a**). Young adults with DS show a plateau in abilities (**d**), while in older adults with DS there is a decrease in raw scores (**e**) likely associated with the development of dementia which results in a further decrease in *z*-scores (**a**)

Figure 1 and Table 4 show relationships between receptive language ability and chronological age, split by sex. Across all ages, *z*-scores decreased with age, and overall, males scored poorer than females. There was no significant interaction between age and sex. For younger and older children, MSEL raw receptive language scores and BPVS3 raw scores respectively increased with age. In younger adults, there was no significant relationship between age and KBIT-2 raw verbal scores, while in older adults, KBIT-2 raw verbal scores decreased with age. No groups showed a significant effect of sex or interaction between age and sex. These results indicate that across the lifespan, receptive language abilities of individuals with DS increasingly deviate from age-typical levels. Within those with DS, these abilities increase in childhood, plateau in young adulthood, then decline in older adulthood (Fig. 1).

**Table 4:** Associations between receptive language ability and age and sex and the interaction between age and sex

	Number (*n*)	Score	Age	Sex	Age*sex
All age groups	523 (271 males, 252 females)	− 3.96 ± 1.33 (− 6.54, 0.14)	F(1,519) = 203.99, *p* < 0.001, *η*_*p*_^2^ = 0.28	*F*(1,519) = 5.89, *p* = 0.016, *η*_*p*_^2^ = 0.01	*F*(1,519) = 2.90, *p* = 0.089, *η*_*p*_^2^ = 0.01
Younger children	104 (59 males, 45 females)	17.01 ± 7.38 (4, 36)	*F*(1,100) = 214.01, *p* < 0.001, *η*_*p*_^2^ = 0.68	*F*(1,100) = 0.28, *p* = 0.595, *η*_*p*_^2^ < 0.01	*F*(1,100) = 2.05, *p* = 0.155, *η*_*p*_^2^ = 0.02
Older children	25 (10 males, 15 females)	62.24 ± 17.48 (33, 106)	*F*(1,21) = 27.31, *p* < 0.001, *η*_*p*_^2^ = 0.57	*F*(1,21) = 3.41, *p* = 0.079, *η*_*p*_^2^ = 0.14	*F*(1,21) = 2.65, *p* = 0.118, *η*_*p*_^2^ = 0.11
Younger adults	157 (80 males, 77 females)	33.48 ± 16.30 (2, 82)	*F*(1,153) = 0.02, *p* = 0.882, *η*_*p*_^2^ < 0.01	*F*(1,153) = 2.16, *p* = 0.144, *η*_*p*_^2^ = 0.01	*F*(1,153) = 1.44, *p* = 0.232, *η*_*p*_^2^ = 0.01
Older adults	237 (122 males, 115 females)	22.22 ± 17.86 (0, 80)	*F*(1,233) = 42.24, *p* < 0.001, *η*_*p*_^2^ = 0.15	*F*(1,233) = 0.75, *p* = 0.388, *η*_*p*_^2^ < 0.01	*F*(1,233) = 0.68, *p* = 0.409, *η*_*p*_^2^ < 0.01

Analyses were performed for receptive language *z*-scores across all age groups, for MSEL raw receptive language scores for younger children, for BPVS3 raw scores for older children, and for KBIT-2 raw verbal scores for younger and older adults. Values for score show mean ± standard deviation (range). Analyses indicated across all ages *z*-scores decreased with age, and were lower for males compared to females. In younger and older children, MSEL raw receptive language scores and BPVS3 raw scores respectively increased with age. In older adults, KBIT-2 raw verbal scores decreased with age

### Cognitive outcomes associated with health comorbidities

For younger children, mean MSEL raw scores ranged from 5.00 to 31.75 with a mean of 17.26 (SD = 6.19). Model 1 explained 72.0% of variance in mean MSEL raw scores. Increased age was significantly associated with increased MSEL raw score, while sex and SES were non-significant predictors (age: unstandardized *B* = 4.57, 95% CI (3.93, 5.21), standardised beta = 0.84, *p* < 0.001). In Model 2, no physical measurements or health comorbidities reliably explained additional variance in MSEL raw scores (Table 5).

**Table 5:** Regression analyses investigating the relationships between health phenotypes and cognitive abilities in younger children (*n* = 99)

	Total *R*^2^	*R*^2^ change	Unstandardised *B* (95% CI)	Standardised beta	*p* value
Height	0.77 ^a^	0.01	− 0.08 (− 0.22, 0.05)	− 0.13	0.216
Weight	0.77 ^a^	< 0.01	− 0.05 (− 0.43, 0.33)	− 0.02	0.802
Head circumference	0.77 ^a^	< 0.01	0.14 (− 0.27, 0.55)	0.05	0.503
Congenital heart defects	0.72	< 0.01	0.34 (− 1.10, 1.78)	0.03	0.639
Congenital heart defects – AVSD only vs none	0.71 ^b^	< 0.01	− 0.01 (− 1.58, 1.57)	> − 0.01	0.992
Reflux	0.72	< 0.01	− 0.76 (− 2.27, 0.75)	− 0.06	0.320
Vision impairments	0.72	< 0.01	0.87 (− 0.88, 2.62)	0.06	0.327
Hearing impairments	0.72	< 0.01	0.21 (− 1.42, 1.84)	0.02	0.799
Otitis media with effusion	0.72	< 0.01	0.66 (− 0.85, 2.16)	0.05	0.389

Sex, age, and a measure of SES were included in Model 1. All results shown give total *R*^2^ for Model 2, *R*^2^ change from Model 1, unstandardized *B* (95% CI), standardised beta, and *p* value for each health phenotype.

*AVSD* atrioventricular septal defect

^a^ Model 1 included age at physical measurement rather than age at medical history telephone interview

^b^ variance explained by Model 1 smaller than for other comorbidities due to a smaller sample; those with a congenital heart defect other than AVSD were excluded from analysis.

For younger adults, KBIT-2 total raw scores ranged from 2 to 108, with a mean of 48.04 (SD = 21.41). Model 1 explained 6.6% of variance in KBIT-2 raw scores. Higher SES was significantly associated with increased KBIT-2 raw score, while age and sex were non-significant predictors (SES: unstandardized *B* = − 3.37, 95% CI (− 5.82, − 0.93), standardised beta = − 0.25, *p* = 0.007). In Model 2 only autism and epilepsy reliably explained additional variance in KBIT-2 raw scores, with the presence of either comorbidity associated with poorer scores (Table 6). Assessing relationships for verbal and non-verbal subscales separately, autism reliably explained additional variance for both verbal and non-verbal scores (verbal: total *R*^2^ = 0.14, *R*^2^ change = 0.06, unstandardized *B* = − 11.56, 95% CI (− 19.64, − 3.48), standardised beta = − 0.24, *p* = 0.005; non-verbal: total *R*^2^ = 0.17, *R*^2^ change = 0.13, unstandardized *B* = − 7.15, 95% CI (− 10.41, − 3.89), standardised beta = − 0.37, *p* < 0.001), while epilepsy reliably explained additional variance for verbal scores only (verbal: total *R*^2^ = 0.12, *R*^2^ change = 0.04, unstandardized *B* = − 10.98, 95% CI (− 20.36, − 1.61), standardised beta = − 0.20, *p* = 0.022; non-verbal: total *R*^2^ = 0.04, *R*^2^ change = 0.01, unstandardized *B* = − 2.27, 95% CI (− 6.28, 1.74), standardised beta = − 0.10, *p* = 0.265).

**Table 6:** Regression analyses investigating the relationships between health phenotypes and cognitive abilities in younger adults (*n* = 157)

	Total *R*^2^	*R*^2^ change	Unstandardised *B* (95% CI)	Standardised beta	*p* value
Height	0.07	< 0.01	0.24 (− 0.25, 0.72)	0.11	0.337
Weight	0.07	< 0.01	0.06 (− 0.19, 0.30)	0.04	0.653
BMI	0.07	< 0.01	< 0.01 (− 0.59, 0.59)	< 0.01	0.995
Head circumference	0.07	< 0.01	− 0.54 (− 2.31, 1.23)	− 0.06	0.546
Autism	0.15	0.09	− 18.72 (− 29.23, − 8.21)	− 0.30	0.001
Depression	0.08	0.01	− 8.19 (− 20.08, 3.70)	− 0.13	0.175
Epilepsy	0.10	0.03	− 13.27 (− 25.72, − 0.82)	− 0.19	0.037
Obstructive sleep apnoea	0.07	< 0.01	0.61 (− 9.80, 11.01)	0.01	0.908
Congenital heart defects	0.09	0.02	6.24 (− 1.31, 13.79)	0.15	0.104
Congenital heart defects—AVSD only vs none	0.12 ^a^	0.03	8.09 (− 1.25, 17.43)	0.18	0.089
Hypothyroid	0.07	< 0.01	0.46 (− 7.80, 8.72)	0.01	0.913
Vision impairments	0.07	< 0.01	4.32 (− 4.63, 13.26)	0.08	0.341
Hearing impairments	0.07	< 0.01	− 2.43 (− 12.57, 7.72)	− 0.04	0.636
Otitis media with effusion	0.08	0.01	− 4.70 (− 13.30, 3.89)	− 0.10	0.281

Sex, age, and a measure of SES were also included in Model 1. All results shown give total *R*^2^ for Model 2, *R*^2^ change from Model 1, unstandardized *B* (95% CI), standardised beta, and *p* value for each health phenotype.

*AVSD* atrioventricular septal defect

^a^ Variance explained by Model 1 larger than for other comorbidities due to a smaller sample; those with a congenital heart defect other than AVSD were excluded from analysis.

## Discussion

We describe the patterns in prevalence rates of multiple physical health and psychiatric comorbidities associated with DS across the lifespan. There were few sex differences in prevalence, though psychiatric comorbidities showed different patterns between males and females with DS relative to population sex differences, with SMRs elevated in males and reduced in females for schizophrenia, bipolar disorder, and anxiety. SMRs indicated rates that were greatly elevated for dementia and also increased for neurodevelopmental comorbidities (autism and ADHD) and depression in both males and females with DS, though differences were more pronounced in females for dementia and autism, and in males for depression. Only age in younger children, and SES, autism, and epilepsy in younger adults, were predictive of cognitive ability. Given our results, we have suggested a number of implications for clinical practice (Table 7).

**Table 7:** Implications for clinical practice

• Clinical guidance tends to be focussed on the needs of children with DS, but the pattern of comorbidities varies across the lifespan and surveillance needs to be adapted accordingly: o Epilepsy is more common in older adults compared to other age groups, and this is likely associated with the development of dementia. o Obstructive sleep apnoea requires on-going surveillance throughout the lifespan. o Thyroid disorders, particularly hypothyroidism, become more common with ageing. o Reflux is a common concern in children with DS. o Hearing and vision problems remain an important consideration throughout life, but these have different causes at different ages. ▪ For hearing, otitis media with effusion is a common issue in childhood, while other causes of hearing loss become important in adulthood. ▪ Vision problems increase across the lifespan, with the increased occurrence of cataracts in adulthood. • Unlike in the typically developing population, most mental health conditions are equally common in males and females, requiring similar surveillance in both sexes to ensure equitable care. • Neurodevelopmental disorders such as autism and ADHD are relatively common and do not show the same sex patterns as in the general population. These should be included in assessment and treatment guidance for all individuals. • To improve cognitive outcomes, a focus on interventions for those with DS from lower SES families and for those with autism or epilepsy is required.	

### Health comorbidities across the lifespan

Rates reported here were largely similar to those reported previously [6–9, 21], though we noted several differences; we did not observe a high prevalence of type 1 diabetes [6, 7] or leukaemia [7], and our finding of increased rates of depression in DS compared to population rates contrasts with Alexander et al. [6] who found a lower incidence in DS using primary care data. Finally, lower rates for epilepsy in younger children contrasts with previous reports [22], possibly due to improved health care, though as with other observational studies, a sampling bias cannot be excluded with parents less willing to participate if children have a severe health condition.

Similar to our results, previous studies have reported increased rates of dementia, epilepsy, hypothyroidism, cataracts, and hearing loss with increased age in individuals with DS [6, 21, 23], and an increased prevalence of otitis media with effusion and congenital heart defects in younger individuals [24, 25]. The change in prevalence across the lifespan for some of these comorbidities in part likely reflects the natural changes associated with development and ageing.

The increased prevalence of autism, ADHD, and obstructive sleep apnoea in younger adults compared to older adults may reflect increased awareness of these comorbidities in younger individuals [10, 11]. For congenital heart defects and related surgery, and for leukaemia, the increased rate in younger adults most likely reflects a cohort effect due to improved care and survival in recent decades.

### Sex differences in prevalence rates of health comorbidities

We found few significant differences in prevalence rates between males and females with DS across the lifespan, with increased prevalence of otitis media with effusion in males for younger children and older adults, reflux in males for younger adults, and hypothyroidism in females for older adults being the only observed differences. In comparison with population rates, dementia, autism, ADHD, and depression were elevated for both males and females with DS, with this relationship more pronounced in females for dementia and autism, and in males for depression. Other than for depression, SMRs suggested higher risk in males with DS, but lower risk in females with DS, for mental illness compared to their peers in the general population. These results indicate altered sex profiles for psychiatric comorbidities in DS relative to the general population, where autism, ADHD, and schizophrenia are more common in males [26, 27], and dementia, bipolar disorder, depression, and anxiety are more common in females [28, 29]. The prevalence rates for these comorbidities did not significantly differ between males and females with DS, suggesting lower modification of risk by sex-related factors compared to that in the general population.

### Receptive language across the lifespan

We explored changes in receptive language ability across the lifespan in DS using both age-adjusted *z*-scores and raw scores. Using *z*-scores to compare to age-typical performance, we found evidence for an increasing divergence from age-typical performance across the lifespan in DS. Examining raw scores showed increases in scores for younger and older children, a plateau in scores for younger adults, and a decrease in scores for older adults, indicating that the deviation from age-typical performance in children is driven by slower development while decline in ability occurs in older adults, likely associated with the high rates of development of dementia. Similarly, Couzens et al. [30] described decreases in standardised scores for cognitive abilities as age increases in those with DS indicating slower development, with raw scores for multiple cognitive abilities increasing over childhood and plateauing in early adulthood. We also found a large degree of variation in ability at any age.

### Cognitive outcomes associated with health comorbidities

In younger children, only age contributed to variance in cognitive ability. In younger adults, SES, autism, and epilepsy contributed to variance in cognitive ability. Similarly, previous studies have reported poorer abilities in those with DS and autism or epilepsy [11, 31]. The mechanisms underlying these relationships in DS are unknown, though it is possible those with multiple neurodevelopmental atypicalities also show an increased vulnerability to a more severe ID. Our finding of higher SES being associated with higher cognitive abilities requires further investigation, and suggests targeting lower SES families for specific interventions as this relationship may occur through increased opportunities for those with higher SES leading to improved cognitive development. However, a shared heritability of genes associated with cognitive abilities cannot be excluded, with parents of individuals with higher SES being more likely to have higher IQs.

In contrast to a previous report, we found no relationship between obstructive sleep apnoea and poorer cognitive abilities [32], though we did not conduct detailed assessments for sleep problems or take account of obstructive sleep apnoea severity. We also did not find a relationship between congenital heart defects and cognitive abilities, suggesting that if appropriately managed, such defects may have no long-term effects on cognitive outcomes despite the potentially deleterious effects of prolonged hospitalisation, anaesthesia, and ischaemic damage. In the typically developing population, congenital heart defects have been associated with poorer cognitive abilities [13]. Previous studies in DS have suggested a similar association in infants and toddlers but not school-aged children [33, 34]. Additional studies are needed to further explore this relationship.

### Strengths and limitations

The strengths of the current study include its large community-dwelling sample, cognitive assessments, and wide age range. Based on UK prevalence data [1], we have recruited approximately 3.5% of younger children and 1.5% of all adults with DS in England and Wales. This allowed us to provide important data on the health comorbidities associated with DS across the lifespan, which may help clinicians with estimating prognosis and providing appropriate care.

Limitations include the possible confounding of age effects with cohort effects, and possible underestimation of the prevalence of some comorbidities. A longitudinal or accelerated longitudinal approach would account for potential cohort effects, taking into account the differences in health and social care for people with DS over the decades, which may impact on the development of certain health conditions. While our study may provide more accurate estimates of prevalence rates than those based on hospital or specialist clinic samples (which are biased towards individuals with more severe conditions), in common with other community surveys, individuals with acute conditions (such as children undergoing treatment for leukaemia) may be under-represented, and medical histories may become less reliable over time for older individuals. Although unlikely, it is also possible that some individuals may have had an undiagnosed health condition, leading to under-estimates of their prevalence. In particular, psychiatric comorbidities may be under-diagnosed and under-recognised in those with DS, though the UK has specialist mental health services for those with ID, indicating our figures are likely to be relatively accurate estimates. Further, if undiagnosed health conditions were subsequently not treated, they may have negatively affected cognitive abilities. In addition, small numbers for some comorbidities resulted in limited power to detect age and sex variations, though when comparing psychiatric comorbidity rates in adults with DS to general population rates, SMRs using the indirect method were employed to account for this as is recommended for rare events. Existing general population data were used for these SMR calculations rather than the collection of new general population data. These differences in collection methods may not account for differences in the medical and psychological attention that individuals with DS and adults in the general population receive. However, as the UK has a comprehensive National Health Service and specialist mental health services for individuals with IDs including those with DS, it is unlikely that this had a significant effect. Finally, our age groups span a number of years, and larger sample sizes would allow more specific age group comparisons to be made.

### Future directions

The altered expression of genes on chromosome 21 and their impact on genomic regulation is thought to be the main factor contributing towards the phenotypes associated with DS, and likely accounts for the difference in sex-related psychiatric profiles compared to the general population. However, due to the variability in phenotypes, genetic variants within chromosome 21 and other chromosomes and environmental factors also have a role. Further, common genetic pathways may influence multiple phenotypes of DS, or there may be direct relationships between phenotypes. Identifying the variability within health comorbidities and factors contributing towards these will help to develop personalised care, and to identify individuals who may be at risk for specific comorbidities to allow for earlier intervention.

## Conclusions

We found that multiple comorbidities show variations in prevalence across the lifespan in DS, and in adults, there are differences in the rates of psychiatric comorbidities for males and females relative to expected population rates, with more pronounced SMRs for dementia and autism in females, and for depression, schizophrenia, bipolar disorder, and anxiety in males. Further, only autism and epilepsy were found to be associated with poorer cognitive outcomes in those aged 16–35 years. Our results provide important information for clinicians to ensure appropriate care and treatment for those with DS, including prognostic information relating to cognitive outcomes in those with comorbidities, and we have provided information on the implications of our findings for clinical practice.

## Supplementary information

**Additional file 1.** Observed counts and rates for psychiatric comorbidities split by sex and age bands

## Data Availability

The datasets used and/or analysed during the current study are available from the corresponding author on reasonable request.

## Abbreviations

ADHD: Attention deficit hyperactivity disorder
AVSD: Atrioventricular septal defect
BMI: Body mass index
BPVS3: British Picture Vocabulary Scale 3
CIs: Confidence intervals
DS: Down syndrome
ID: Intellectual disability
KBIT-2: Kaufmann Brief Intelligence Test 2
MSEL: Mullen Scales of Early Learning
OR: Odds ratio
SES: Socioeconomic status
SMRs: Standardised morbidity ratios
