# Supplementary material for: Health comorbidities and cognitive abilities across the lifespan in Down syndrome
Source: J Neurodev Disord. 2020 Jan 23;12:4. doi: 10.1186/s11689-019-9306-9 (PMC6979347; doi:10.1186/s11689-019-9306-9)
Supplement: Supplementary file 1 — Additional file 1. Observed counts and rates for psychiatric comorbidities split by sex and age bands [file 11689_2019_9306_MOESM1_ESM.docx]

|  | **Males** | | | | | | **Females** | | | | | |
| --- | --- | --- | --- | --- | --- | --- | --- | --- | --- | --- | --- | --- |
|  | **16-25 years** | **26-35 years** | **36-45 years** | **46-55 years** | **56-65 years** | **66-75 years** | **16-25 years** | **26-35 years** | **36-45 years** | **46-55 years** | **56-65 years** | **66-75 years** |
| **Total** | 44 | 39 | 44 | 73 | 27 | 9 | 45 | 42 | 32 | 68 | 27 | 2 |
| **Autism** | 9 | 4 | 3 | 1 | 2 | 0 | 5 | 5 | 1 | 0 | 0 | 0 |
| **ADHD** | 2 | 1 | 0 | 0 | 0 | 0 | 1 | 1 | 1 | 0 | 0 | 0 |
| **Schizophrenia** | 0 | 0 | 1 | 1 | 0 | 1 | 0 | 1 | 0 | 0 | 0 | 0 |
| **Bipolar disorder** | 0 | 0 | 1 | 2 | 0 | 1 | 0 | 1 | 1 | 0 | 1 | 0 |
| **Depression** | 2 | 6 | 11 | 10 | 3 | 2 | 3 | 10 | 6 | 14 | 6 | 0 |
| **Anxiety** | 4 | 6 | 4 | 4 | 4 | 0 | 2 | 2 | 4 | 2 | 0 | 0 |
| **Dementia** | 0 | 0 | 3 | 21 | 14 | 5 | 0 | 0 | 5 | 25 | 16 | 1 |

**Supplementary Table 1a. Observed counts for adults with DS and total sample sizes for psychiatric comorbidities split by sex and age bands.** ADHD – attention deficit hyperactivity disorder.

|  |  | Males | | | | | | | Females | | | | | | |
| --- | --- | --- | --- | --- | --- | --- | --- | --- | --- | --- | --- | --- | --- | --- | --- |
|  |  | 16-25 years | 26-35 years | 36-45 years | 46-55 years | 56-65 years | 66-75 years | Total | 16-25 years | 26-35 years | 36-45 years | 46-55 years | 56-65 years | 66-75 years | Total |
| Autism | DS | 156·63 | | 34·19 | | 55·56 | | 246·37 | 114·94 | | 10·00 | | 0·00 | | 124·94 |
|  | Population | 22·4 | | 2 | | 11·7 | | 36·1 | 7·1 | | 0 | | 0 | | 7·1 |
| ADHD | DS | 45·45 | 25·64 | 0·00 | 0·00 | 0·00 | 0·00 | 71·10 | 22·22 | 23·81 | 31·25 | 0·00 | 0·00 | 0·00 | 77·28 |
|  | Population | 3·4 | 3·5 | 2·1 | 2·3 | 2 | 0·8 | 14·1 | 3·2 | 2·1 | 2·7 | 2·6 | 2·1 | 1·2 | 13·9 |
| Schizophrenia | DS | 0·00 | 0·00 | 22·73 | 13·70 | 0·00 | 111·11 | 147·54 | 0·00 | 23·81 | 0·00 | 0·00 | 0·00 | 0·00 | 23·81 |
|  | Population | 2·1 | 8·6 | 11·6 | 6·5 | 8·7 | 2·7 | 40·2 | 7·4 | 10·7 | 11·3 | 9·8 | 6·4 | 3·1 | 48·7 |
| Bipolar disorder | DS | 0·00 | 0·00 | 22·73 | 27·40 | 0·00 | 111·11 | 161·24 | 0·00 | 23·81 | 31·25 | 0·00 | 37·04 | 0·00 | 92·10 |
|  | Population | 31·3 | 31 | 29 | 20·6 | 16·3 | 3·6 | 131·8 | 37·3 | 31 | 19 | 12·4 | 13·3 | 4·3 | 117·3 |
| Depression | DS | 45·45 | 153·85 | 250·00 | 136·99 | 111·11 | 222·22 | 919·62 | 66·67 | 238·10 | 187·50 | 205·88 | 222·22 | 0·00 | 920·37 |
|  | Population | 9 | 41 | 27 | 42 | 42 | 24 | 185 | 38 | 28 | 55 | 48 | 44 | 19 | 232 |
| Anxiety | DS | 90·91 | 153·85 | 90·91 | 54·79 | 148·15 | 0·00 | 538·61 | 44·44 | 47·62 | 125·00 | 29·41 | 0·00 | 0·00 | 246·48 |
|  | Population | 38 | 60 | 68 | 60 | 62 | 20 | 308 | 90 | 63 | 70 | 85 | 67 | 58 | 433 |
| Dementia | DS | 0·00 | 0·00 | 68·18 | 287·67 | 518·52 | 555·56 | 1429·93 | 0·00 | 0·00 | 156·25 | 367·65 | 592·59 | 500·00 | 1616·49 |
|  | Population | 0 | 0 | 0 | 0 | 9 | 24 | 33 | 0 | 0 | 0 | 0 | 9 | 23 | 32 |

Supplementary Table 1b. Rates for adults with DS and the general population for psychiatric comorbidities split by sex and age bands. Figures show rates per 1000, aside from total rates, which are summed from the previous columns and are therefore rates per 6000. Observed rates for adults with DS were determined based on our observed counts and sample sizes (Supplementary Table 1a), and converted to the equivalent figure per 1000. Expected rates for the general population were determined based on published rates and converted to the equivalent figure per 1000 (rates from Prince et al [18] for dementia and from McManus et al [19] for all other comorbidities). ADHD – attention deficit hyperactivity disorde
